# Supplementary material for: PVDF/Graphene Composite Nanoporous Membranes for Vanadium Flow Batteries
Source: Membranes (Basel). 2019 Jul 19;9(7):89. doi: 10.3390/membranes9070089 (PMC6680472; doi:10.3390/membranes9070089)
Supplement: Supplementary file 1 [file membranes-09-00089-s001.pdf]

PVDF/Graphene Composite Nanoporous Membranes for Vanadium Flow Batteries

Yiming Lai, Lei Wan and Baoguo Wang *

The state key laboratory of Chemical Engineering, Department of Chemical Engineering, Tsinghua University, Beijing 100084, China

* Correspondence: bgwang@mail.tsinghua.edu.cn; +86-10-62788777

Table S1. Influence of SAS contents on PVDF nanoporous membranes.

SAS Contents (wt %)	Thickness (μm)	Permeability of H^+ (×10^{-5} $cm^2 \cdot min^{-1}$)	Permeability of VO^{2+} (×10^{-7} $cm^2 \cdot min^{-1}$)	Selectivity	Proton Conductivity ($mS \cdot cm^{-1}$)
10	140	0.36	0.21	175.2	1.6
15	110	1.14	0.99	114.6	10.0
20	124	3.19	5.07	62.9	20.3
25	123	3.41	12.8	26.6	23.5

Table S2. Water uptakes and proton conductivities of the membranes.

Sample	Thickness (μm)	Water Uptake (%)	Area Resistance ($\Omega \cdot cm^2$)	Proton Conductivity (mS/cm)
PVDF	123	29.1	0.524	23.5
PVDF/G-0.05	124	34.2	0.396	31.3
PVDF/G-0.10	141	33.8	0.451	31.3
PVDF/G-0.15	125	32.3	0.337	37.1
PVDF/G-0.2	115	34.6	0.352	32.6
PVDF/G-0.3	128	38.7	0.417	30.7

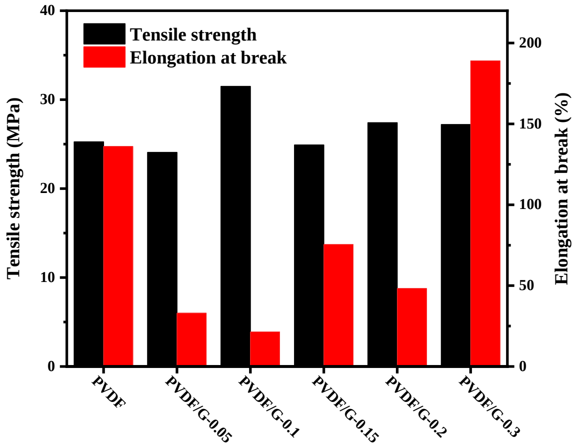

Figure S1. Mechanical properties of PVDF and PVDF/G-0.15 membranes.
